# Timely access to secondary pediatric services in Korea: a key to reducing child and adolescent mortality

**DOI:** 10.4178/epih.e2024059

**Published:** 2024-07-05

**Authors:** Minku Kang, Young June Choe, Hye Sook Min, Saerom Kim, Seung-Ah Choe

**Affiliations:** 1 Department of Preventive Medicine, Korea University College of Medicine, Seoul, Korea; 2 Department of Pediatrics, Korea University College of Medicine, Seoul, Korea; 3 Research Institute of Public Healthcare, National Medical Center, Seoul, Korea; 4 Department of Preventive Medicine, Inje University College of Medicine, Busan, Korea; 5 Research and Management Center for Health Risk of Particulate Matter, Korea University, Seoul, Korea

**Keywords:** Pediatrics, Child, Adolescent, Mortality

## Abstract

**OBJECTIVES:**

Geographic disparities in access to secondary pediatric care remain a significant issue in countries with universal health coverage, including Korea. This study investigated the link between geographic access to secondary pediatric care and mortality rates in children and adolescents (0-19 years) in Korea.

**METHODS:**

We analyzed district-level data to assess the percentage of those aged 0-19 years residing outside of a 60-minute travel radius from the nearest secondary pediatric care provider (accessibility vulnerability index, AVI).

**RESULTS:**

The AVI ranged from 0% to 100% across the districts for the study period. The confidence interval (CI) was -0.30 (95% CI, -0.41 to -0.19) in 2017 and -0.41 (95% CI, -0.52 to -0.30) in 2021, indicating that the proportion of those who could not access care within 60 minutes was disproportionately higher in districts with lower socioeconomic status. We found 8% rise in mortality rates among individuals aged 0-19 years for every 10% increase in AVI (95% CI, 1.06 to 1.10).

**CONCLUSIONS:**

The study highlights disparities in pediatric care access and their impact on child survival, emphasizing the need for improved access to achieve true universal health coverage.

## GRAPHICAL ABSTRACT

[Fig f2-epih-46-e2024059]

## Key Message

The study investigated the link between geographic access to pediatric services and child mortality in Korea, finding that limited access, particularly during the COVID-19 pandemic, was associated with higher mortality. The research highlights the need for improved access to timely care to reduce regional disparities in preventable deaths among children and adolescents.

## INTRODUCTION

Access to pediatric care is a crucial indicator of a healthcare system’s effectiveness in providing timely and adequate services for children. Many high-income countries face barriers to such care, including limited regional availability and high treatment costs [1]. Korea, in particular, grapples with a shortage of pediatric critical care, which has resulted in the redirection of secondary pediatric services away from underserved rural areas [2]. This shift, which has taken place despite universal health coverage, has raised concerns about an increased risk of preventable child mortality. Therefore, this study aimed to explore the link between geographic access to pediatric services and child and adolescent mortality rates in Korea.

## MATERIALS AND METHODS

We used the merged medical service provision database and national mortality data based on the district code and year. We utilized the yearly accessibility vulnerability index (AVI) in 2017-2021, obtained from the Health Map (http://www.healthmap.or.kr/) [3]. The AVI measures the percentage of individuals aged 0-19 in a district who reside outside of a 60-minute travel radius from the nearest secondary pediatric care provider [3]. We linked this data with mortality statistics for children aged 0-19, creating a multi-year database comprising annual age-specific mortality rates for all 250 districts. To gauge the extent of socioeconomic inequality in AVI across districts, we employed the concentration index, which is characterized as two times the area between the concentration curve and the line of equality (i.e., the 45° line) [4]. A negative concentration index indicates that the outcome of interest is more prevalent among the economically disadvantaged population. We calculated the relative risk (RR) for mortality per 10% increase in the AVI, utilizing generalized estimating equation models that included annual trends, district-level wages, and average salary income tax data from each district. Considering the coronavirus disease 2019 (COVID-19) pandemic, we further stratified the risk estimates based on 2 periods (before 2020 and in 2020 and beyond). Statistical analyses were performed using R version 4.2.0 (R Foundation for Statistical Computing, Vienna, Austria), with a significance level set at p-value=0.05.

### Ethics statement

This study did not involve human subjects and thus did not require institutional review board approval.

## RESULTS

**Figure 1.**
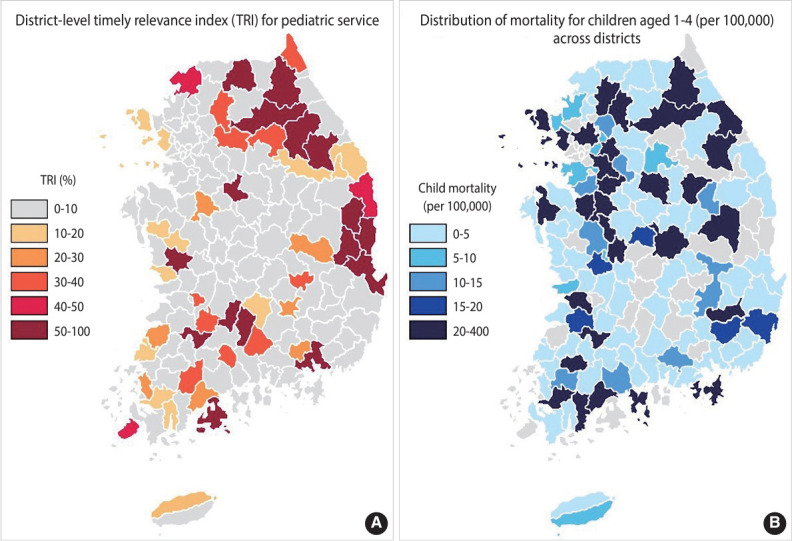
District-level accessibility vulnerability index (AVI) for secondary pediatric services (A, numbers in %) and the distribution of mortality for children aged 1-4 per 100,000 across districts (B) in 2021. The AVI for secondary pediatric care services is defined as the percentage of individuals aged 0-19 in the district who reside outside of a 60-minute travel radius from the nearest secondary pediatric care provider.

During the observed period, the overall mean AVI for pediatric services was 10.4% in 2017 and 10.1% in 2021, and this indicator ranged from 0% to 100% across the districts. In 2017, the concentration index for the AVI was -0.30 (95% confidence interval [CI], -0.41 to -0.19), and in 2021, it was -0.41 (95% CI, -0.52 to -0.30), indicating a higher proportion of individuals unable to access secondary pediatric care within 60 minutes in districts with lower socioeconomic status. While the national average mortality rate for individuals aged 0-19 decreased from 25.7 per 100,000 in 2017 to 20.8 per 100,000 in 2021, the difference between the highest and lowest district mortality increased from 93 to 126 per 100,000. The AVI and child mortality showed similar patterns of distribution (Figure 1). We observed an 8% rise in the mortality rate when AVI increased by 10% (95% CI, 1.06 to 1.10). This association remained consistent across all age groups except for infants, with its highest peaks among those aged 5-9 years and 10-14 years. The mortality risk was greater during the pandemic (RR, 1.11; 95% CI, 1.08 to 1.14) than before it (RR, 1.07; 95% CI, 1.05 to 1.09, p for heterogeneity=0.016) (Table 1).

## DISCUSSION

We observed consistent socioeconomic disparities in secondary pediatric care access and a worrisome association between limited access and higher child and adolescent mortality, particularly during the COVID-19 pandemic. These results emphasize the need for timely care access to reduce regional disparities in preventable mortality among children and adolescents. Several prior studies worldwide, regardless of healthcare system type, have shown similar findings [5,6].

Previous research has predominantly focused on examining the impact of geographic accessibility on child mortality in remote or rural areas and has shown findings similar to those of our study. In a study of 21 low-income and middle-income countries, travel time to the nearest facility ≥ 20 minutes was associated with a higher risk of neonatal mortality [7]. In Malawi, each additional kilometer to the nearest facility was associated with a 1% increase in the hazard of mortality in under-5 children [8]. The association was close to null in a Kenyan study, conducted in an area characterized by a high density of health facilities [9]. Our discovery contributes additional evidence supporting the positive correlation between the geographical accessibility of secondary pediatric healthcare providers and pediatric mortality.

In addition to transportation accessibility, it is crucial to consider indicators such as car ownership rates and access to public or private transportation, such as ambulances, taxis, or buses. Unfortunately, our dataset lacked this specific information. Nonetheless, previous studies have indicated that distances of 72-80 kilometers or travel times exceeding 60 minutes to specialist healthcare services can serve as indicators of health vulnerability [5,10]. We believe that the AVI can still serve as a valid indicator for assessing the accessibility of care in the pediatric population.

The observation of a stronger association during the COVID-19 pandemic suggests that the social context can also affect the impact of geographical access to care. While our findings suggest the importance of geographic access to care, individual-level data are needed for validation. In the context of universal health coverage, improving access to secondary pediatric care can narrow the child health gap.

## Figures and Tables

**Figure f2-epih-46-e2024059:**
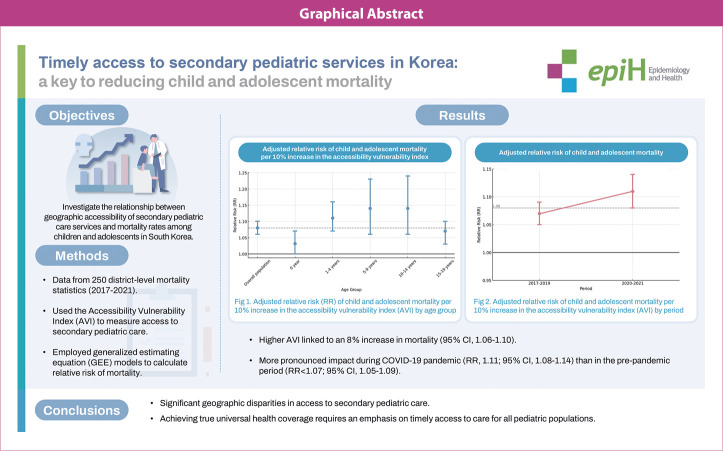


**Table 1.** Adjusted RR of child and adolescent mortality per 10% increase in the AVI for secondary pediatric services^1^

Study population and period	RR (95% CI)
Overall population	1.08 (1.06, 1.10)
Age (yr)	
0	1.03 (1.00, 1.07)
1-4	1.11 (1.07, 1.16)
5-9	1.14 (1.06, 1.23)
10-14	1.14 (1.06, 1.24)
15-19	1.07 (1.03, 1.10)
Period	
2017-2019	1.07 (1.05, 1.09)
2020-2021 (COVID-19 pandemic)	1.11 (1.08, 1.14)

RR, relative risk; CI, confidence interval; AVI, accessibility vulnerability index; COVID-19, coronavirus disease 2019.

1 The RR is adjusted for year and annual salary income tax, which is a proxy for the economic status of the district; Risk estimates and CIs were calculated per 10% increase in AVI.
